# Implementation and Effects of Information Technology-Based and Print-Based Interventions to Promote Physical Activity Among Community-Dwelling Older Adults: Protocol for a Randomized Crossover Trial

**DOI:** 10.2196/15168

**Published:** 2020-04-27

**Authors:** Claudia R Pischke, Claudia Voelcker-Rehage, Manuela Peters, Tiara Ratz, Hermann Pohlabeln, Jochen Meyer, Kai von Holdt, Sonia Lippke

**Affiliations:** 1 Institute of Medical Sociology Centre for Health and Society Medical Faculty, University of Duesseldorf Duesseldorf Germany; 2 Institute of Human Movement Science and Health Chemnitz University of Technology Chemnitz Germany; 3 Leibniz Institute for Prevention Research and Epidemiology – BIPS Bremen Germany; 4 Jacobs University Bremen Bremen Germany; 5 OFFIS – Institute for Information Technology Oldenburg Germany

**Keywords:** physical activity, older adults, eHealth, print intervention, IT-based intervention, physical activity promotion, healthy aging, preferences, randomized trial

## Abstract

**Background:**

Despite the known health benefits of physical activity (PA), less than half and less than one-third of older adults in Germany reach the PA recommendations for endurance training and strength training, respectively, of the World Health Organization. The aim of this study is to investigate the implementation and effectiveness over the course of 9 months of two interventions (information technology [IT]-based vs print-based) for PA promotion among initially inactive older adults in a randomized, crossover trial. This study is part of a large research consortium (2015-2021) investigating different aspects of PA promotion. The IT-based intervention was previously developed and refined, while the print-based intervention was newly developed during this funding phase.

**Objective:**

We aim to compare the effectiveness and examine the preferences of study participants regarding both delivery modes.

**Methods:**

Our target sample size was 390 initially inactive community-dwelling older adults aged ≥60 years at baseline (3-month follow-up [T1]: expected n=300; 9-month follow-up [T2]: expected n=240) who were randomized to one of two interventions for self-monitoring PA: IT-based (50%) or print-based (50%) intervention. In addition, 30% of the IT-based intervention group received a PA tracker. At T1, participants in both groups could choose whether they prefered to keep their assigned intervention or cross over to the other group for the following 6 months (T2). Participants’ intervention preferences at baseline were collected retrospectively to run a post hoc matched-mismatched analysis. During the initial 3-month intervention period, both intervention groups were offered weekly group sessions that were continued monthly between T1 and T2. A self-administered questionnaire and 3D accelerometers were employed to assess changes in PA between baseline, T1, and T2. Adherence to PA recommendations, attendance at group sessions, and acceptance of the interventions were also tracked.

**Results:**

The funding period started in February 2018 and ends in January 2021. We obtained institutional review board approval for the study from the Medical Association in Bremen on July 3, 2018. Data collection was completed on January 31, 2020, and data cleaning and analysis started in February 2020. We expect to publish the first results by the end of the funding period.

**Conclusions:**

Strategies to promote active aging are of particular relevance in Germany, as 29% of the population is projected to be ≥65 years old by 2030. Regular PA is a key contributor to healthy aging. This study will provide insights into the acceptance and effectiveness of IT-based vs print-based interventions to promote PA in initially inactive individuals aged ≥60 years. Results obtained in this study will improve the existing evidence base on the effectiveness of community-based PA interventions in Germany and will inform efforts to anchor evidence-based PA interventions in community structures and organizations via an allocation of permanent health insurance funds.

**Trial Registration:**

German Registry of Clinical Trials DRKS00016073; https://tinyurl.com/y983586m

**International Registered Report Identifier (IRRID):**

DERR1-10.2196/15168

## Introduction

In Germany, approximately 91% of all deaths are attributable to noncommunicable diseases [1]. Physical inactivity is the fourth leading risk factor contributing to the development of noncommunicable diseases and overall mortality [2]. Conversely, regular physical activity (PA) and the reduction of an inactive lifestyle [3] are associated with improvements in physical, cognitive, and functional health over the lifespan [3,4].

The World Health Organization (WHO) and American College of Sports Medicine recommend that adults aged 18 to 64 years, as well as those 65 years and older, should perform moderate-to-vigorous endurance training for at least 150 minutes per week (in bouts of at least 10 minutes) [2]. In addition, adults aged 65 years and older should perform flexibility, strength, and balance training twice per week [5,6]. Furthermore, Rütten and Pfeiffer [7] state in the German national recommendations for PA that adults should generally avoid extended periods of sitting and, where possible, take PA breaks from sitting. According to the authors, “a major health benefit of PA can already be observed when persons who were entirely physically inactive start becoming active to a small extent meaning that every increase in PA is associated with a health benefit. Every single step away from sedentary behavior is important and promotes health” [7].

In Germany, only 42% of adults aged 65 years and older currently meet the recommendations for endurance (at least 150 minutes per week of moderate activity, at least 75 minutes of intense activity, or a combination of both, in bouts of at least 10 minutes), and 29% meet the recommendations for strength training [6], indicating a need for interventions for PA promotion targeting the general population of older adults. An earlier survey spanning the years 2008 to 2011 reported that merely 18% of German adults aged 60 to 69 years and 14% of adults aged 70 to 79 years exercised moderately to vigorously for at least 150 minutes per week [8]. To tackle this public health issue in Germany and facilitate PA promotion, the Federal Ministry of Health recommends the development and implementation of population-based informational intervention approaches or campaigns, community-based interventions, and policy and environmental approaches for PA promotion [7]. According to the current state of research, results of several reviews suggest that interventions for PA promotion, including mass media campaigns, motivational decision aids, community-based multicomponent interventions, and environmental approaches, can effectively increase PA in the general population [7]. Evidence regarding the benefit of using theory during intervention development to achieve greater impact on behavior change is still contradictory [9]. On the one hand, there is some evidence suggesting that interventions based on behavior change techniques rooted in theory are effective in altering behavior [9]. On the other hand, results of one meta-analysis indicate that a theoretical basis leads to no difference in intervention effectiveness [10], maybe also due to the fact that the behavior preferences of study participants were not sufficiently taken into account [11]. Theories and theoretical assumptions about mechanisms, processes, and techniques are needed to better understand individuals and their needs and to avoid always reinventing the wheel when developing interventions but rather building on previous evidence [3,9,10].

Multiple studies have investigated the role of different modalities of delivering these interventions to older adults. Evidence suggests that participation in interventions providing information on PA either in a face-to-face setting or as print versions leads to increased PA levels in older adults [12-14]. Information technology (IT)-based PA interventions have the advantage of potentially reaching a large number of people in a cost-effective manner [15]. In addition, intervention material can be easily accessed, and instantaneous feedback on behavior change can be provided [10,15]. They also appear to have a positive impact on PA [3,16]. Results of a systematic review comparing the effectiveness of electronic health (eHealth) interventions promoting PA in older adults aged 55 years and older with either no intervention or a non-eHealth intervention indicate that eHealth interventions can effectively promote PA in this population in the short-term, while evidence regarding long-term effects is still lacking [3,9,17]. The results of this review are inconclusive regarding the question of whether eHealth interventions have a greater impact on PA behavior among older adults than non-eHealth (eg, print-based) interventions [17]. Also, the effects of combining various eHealth intervention components are still unclear.

Two studies investigated the added benefit of using Fitbit activity trackers in addition to a website to monitor PA on total weekly PA levels and moderate-to-vigorous PA (MVPA) [18,19]. In a sample of overweight adults, Vandelanotte et al [18] demonstrated a significant increase in total weekly PA and MVPA after 3 months for a group of participants using a Fitbit compared with a group of participants not using a Fitbit. Although sitting time decreased over time, this effect was not significant [18]. With a sample of older adults, Muellmann et al [19] found no significant differences in PA levels between a group using a Fitbit compared with a group not using a Fitbit. However, participants in the Fitbit group had slightly greater increases in MVPA and decreases in sedentary time than participants in the non-Fitbit group after 3 months [19]. While more participants in the non-Fitbit group did not complete the follow-up after 3 months than in the Fitbit group (63% vs 36%) in the first study [18], the reverse was true in the second study [19]. More participants in the Fitbit group did not complete the 3-month follow-up than in the non-Fitbit group (40% vs 31%) [19]. It is conceivable that individual preferences for interventions may have influenced dropout and possibly adherence to the interventions [10,11,13]. Participants may have been randomized to an intervention that they would not have picked had they been given a choice and that they may have found difficult to interact with.

The effects of study participants’ preferences on treatment or intervention outcomes in randomized controlled trials are still not well understood [20,21]. There is some indication that the preferences for delivery mode vary by the sociodemographic characteristics of participants, such as age, gender, and living environment, or by weight status [13,22]. For example, preference for an IT-based intervention was positively related with being 35 years or older (compared to younger ages) and high levels of internet use and was negatively associated with female gender. Preference for a print-based intervention was associated with older age and negatively associated with female gender and obesity [22]. Further, evidence suggests that sociodemographic variables may explain variations in the use of PA trackers [23] and that use of trackers in PA interventions should be aligned with preferences of different target groups [13].

Therefore, the main aim of this study was to compare the effects of 2 interventions using different modalities (IT-based vs print-based) among initially inactive older adults aged at least 60 years living in 14 community districts in 2 geographically different regions (northwest and northeast) of the city of Bremen, Germany. Further, participants’ preferences regarding the intervention modality were addressed by conducting a randomized trial with a crossover design. At baseline, participants were randomized to either a 10-week IT-based or print-based intervention. A random subsample of the IT-based intervention group (30%) also received a PA tracker. After the 3-month follow-up, participants could choose whether they wanted to remain in the same intervention group and keep their assigned material or switch to the other group for the following 6 months. They could also choose to use a PA tracker at this stage.

## Methods

The study is embedded in the larger Physical Activity and Health Equity: Primary Prevention for Healthy Ageing (AEQUIPA) research network that is funded by the Federal Ministry of Education and Research (BMBF) [24]. The AEQUIPA research network is comprised of six subprojects and conducts theory-based and participatory empirical research on different aspects of PA and healthy aging in the northwestern part of Germany [24]. One of the aims of the network is to develop, implement, and evaluate PA interventions for the primary prevention of chronic diseases in adults aged at least 65 years. The first 3-year funding phase was completed in January 2018 [25]. The topic of the research remains the same in the second funding phase (2018-2021); however, in this phase, additional aims are to intensify community participation to reach physically inactive adults, use appropriate technology for PA promotion in the population of older adults, and disseminate and transfer PA interventions as well as results of both funding phases. This study (PROMOTE II) is one of the 6 subprojects of the entire network and builds on knowledge gained during the preceding study (PROMOTE I) [19,25,26].

In the previous study, the research aims were to develop and test 2 individually tailored IT-based interventions for the promotion of a physically active lifestyle in adults aged 65-75 years in a community-based intervention trial [25]. Intervention effects on physical, psychological, and cognitive indicators for healthy aging were examined by comparing 2 intervention groups to a delayed-intervention control group [26]. Results of this trial are reported elsewhere [19,26]. Briefly, the proportion of already active intervention participants at baseline was relatively high. At follow-up, they reported increased social-cognitive predictors for behavior change, but no significant increases in the primary outcome of MVPA when compared with the control group. Also, the dropout rate was higher in the group invited to use PA trackers in addition to the IT-based intervention compared with the IT-only intervention suggesting that individuals with little technological experience might have been randomized to an intervention that they found too difficult to use. In addition, participants in both intervention groups requested printed diaries to track their PA during times that they had limited access to computers. Thus, reactions of the target population during the first funding phase suggested that a proportion of participants prefers a print-based intervention or would appreciate an app for accessing a PA diary on their smartphone or tablet.

### Study Aims

Hence, based on previous findings of the literature and knowledge gained during the preceding trial, this study aimed to adapt and simplify the IT-based intervention of the first funding phase further to improve usability and develop a simple print-based intervention that initially inactive participants with little affinity to technology find easy to interact with; investigate the implementation, feasibility, and use of 2 interventions (IT-based vs print-based) as well as changes in PA among older adults (aged ≥60 years) in a randomized trial with a crossover design over the course of 9 months; examine the role of personal preferences for different delivery modes with regard to intervention effectiveness; and explore associations between changes in PA and possible changes in physical fitness and cognitive capacity in a pooled sample of participants from both phases of funding.

### Selection of Communities for the Study, Participants, and Procedures

We selected 14 community districts in 2 geographically different regions (northwest and northeast) of the city of Bremen, Germany: Burgdamm, Lesum, St. Magnus, Vegesack, Schönebeck, Aumund-Hammersbeck, Rönnebeck, Radio Bremen, Riensberg, Gartenstadt Vahr, Neue Vahr Südwest, Oberneuland, Ellener Feld, and Blockdiek. They were chosen because there are study centers in these districts that can be easily reached by the target group and the project team had already established prior liaisons with stakeholders in these areas of the city who could facilitate community involvement in the implementation of the intervention.

Names and addresses of men and women aged ≥60 years residing in the chosen community districts were drawn from the records of the residents’ registration office. Subsequently, they were invited to participate in the study via mail. Reminders were sent out in case of no response after 2 weeks. The study was also publicized in local newspaper articles and mentioned during talks that targeted older adults by researchers in the team. Individuals made aware of the study through this channel could contact the research team directly to be screened for eligibility. Eligibility for study participation was determined during computer-assisted telephone interviews with trained study nurses based on the inclusion and exclusion criteria.

### Inclusion and Exclusion Criteria

Men and women were eligible for study participation if they were aged ≥60 years (there was no upper age limit), lived independently (ie, in own apartment or room without assisted living or regular home nursing), and provided informed consent to participate in the study. Additional inclusion criteria were basic knowledge of German, the ability to walk without a walking aid and participate in study assessments and weekly group meetings without external assistance, and no planned long absence (ie, for more than 2 weeks). Another precondition was the availability of a device with internet access in the household, regular access to the device, and the ability to use it.

Individuals were excluded from the study if they reported that they had already been regularly physically active for more than 1 year and had reached the intervention target (ie, the recommended 150 minutes of moderate-intensity endurance training per week). They were also excluded if they reported having participated in the preceding study (PROMOTE I), had planned a vacation lasting longer than 2 weeks during the intervention period, had been medically prohibited to be physically active, displayed severe visual or cognitive impairment (Mini-Mental-State Examination Score ≤14) or other impairments (eg, due to a stroke, transient ischemic attack, brain surgery, or neurological diseases, such as Alzheimer’s disease, multiple sclerosis, or Parkinson’s disease), had an implanted device (eg, pacemaker, implanted hearing aid), or experienced occasional syncope. Additional exclusion criteria were a lack of medical clearance because of fractures or surgeries in the past 6 months that could potentially constrain study participation; report of severe diseases of the cardiovascular system (eg, cardiac arrhythmia, heart failure, or nonmedicated hypertension) or respiratory system (eg, COPD, severe asthma); or severe limitations due to arthritis or osteoarthritis in the legs or severe osteoporosis or acute injuries of the spine. Individuals with diabetes diagnosed less than 6 months prior and without medical clearance were also excluded from the study.

### Study Design

**Figure 1:**
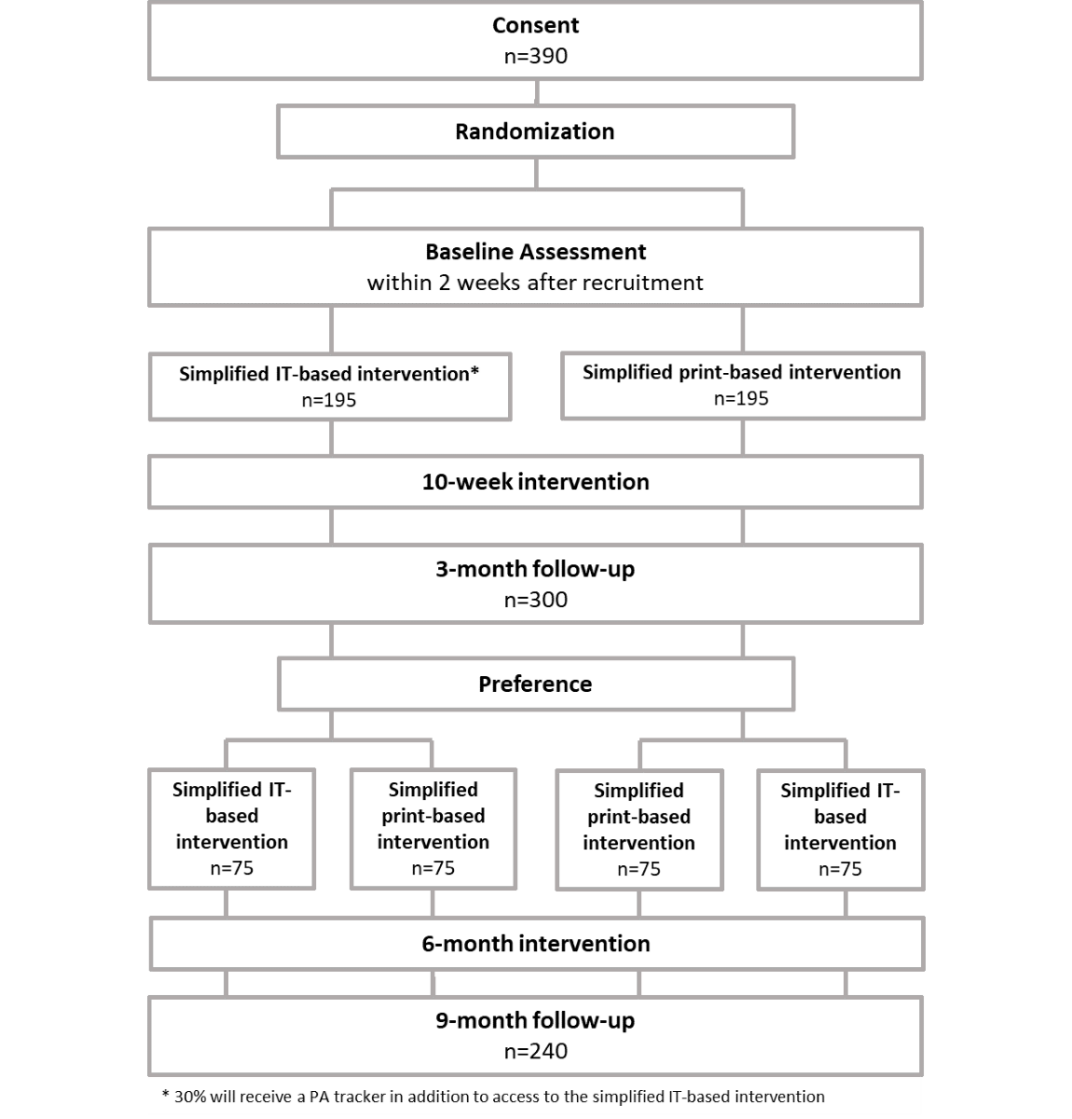
Study design.

After determination of eligibility, study participants were randomized to either an IT-based intervention or a print-based intervention (see Figure 1). In the first group, 30% were randomly selected to receive a PA tracker in addition to access to the website. Weekly time slots were randomly assigned to both intervention groups. Then, we had participants choose appointments according to their personal time constraints without knowing which intervention group they were assigned to. All intervention groups were offered weekly group sessions, and participants were encouraged to attend each of them. Two weeks before the intervention started, participants in all groups attended an introductory event where they were informed about the study background and procedures. On this day, data collection also started, and participants received the activity tracker and questionnaires for the first time (T0). They were also briefed about their randomization to one of the intervention groups and the existence of the other groups. Therefore, they were not blinded. Participants were given the choice to remain in their intervention group or to cross over to the other group after 3 months (and a 10-week intervention period). With that, a crossover design with the following possible combinations was generated: IT-IT, print-print (matched), or IT-print and print-IT. Preferences regarding the intervention material assessed retrospectively at the 9-month follow-up will be taken into account when analyzing intervention effects later in the study. Research staff members conducting the study were not blinded, and the statistician will not be blinded when analyzing the data.

### Analytic Strategy

Effects of time and group (main outcomes: subjective and objective measures of PA) will be examined in multivariate analyses. In addition, covariates, such as gender, age, and preferences regarding intervention material, and individual contextual factors (eg, living or social environment) will be taken into account. Furthermore, we will analyze how the intervention enables study participants to change their behavior by running mediation analyses. The estimated target sample size of 390 participants for this study is based on the results of 2 recently published studies on PA in adults [27,28] that reported a mean 111 weekly minutes (SD 116.8 weekly minutes) of MVPA and an intervention effect of an increase in MVPA of 77 minutes per week. However, in a conservative (or realistic) approach, we assumed that the mean increase in MVPA in our study will reach only 40 minutes/week. To detect such a difference between the intervention and control groups with a power of 80% at an alpha of .05 (two-sided), a net sample size of 150 subjects for each group is required. Assuming a loss to follow-up from T0 to T1 of 20-25% (ie, ca. 23%), it is necessary to initially recruit 390 study participants (ie, 195 per group at baseline). Assuming another 20% loss to follow-up from T1 to T2, we expect that approximately 240 individuals at T2 will self-select to one of the 4 groups: IT-IT, print-print, IT-print, or print-IT. Should participants self-select equally into each group (n=60), we will still be able to detect the mean difference of 77 minutes/week [28] between each group, by means of an appropriate post hoc test that is adjusted for multiple comparisons.

### Measures

The primary outcome is change in PA, which is assessed at baseline, 3 months, and 9 months using triaxial accelerometers worn at the right hip during the day over the course of 7 days and a self-administered questionnaire. Participants received the self-administered questionnaire and accelerometer at their introductory event at baseline (T0), after 3 months (T1) at their tenth group meeting, and after 9 months (T2) at their last monthly meeting. Further, due to cost issues, a random subsample of 114 participants (equally distributed across the intervention groups) underwent additional anthropometric, physical fitness, and cognitive tests to assess secondary outcomes at all 3 time points. Physical fitness was assessed using gait speed via a 4-meter walk test [29], handgrip strength using a dynamometer [30], and cardiovascular fitness using the 2-minute step test [31]. Additionally, participants’ weight was assessed using a scale. Attention and inhibition were assessed using a cognitive test (Simon task) [32].

Data from the objective measures of physical fitness and cognitive performance (Mini Mental State Examination and executive function test) will be preprocessed using customized MATLAB routines and analyzed using multivariate generalized linear models. Data for physical fitness and cognitive performance assessed at baseline and after the 10-week intervention during the first funding phase and in the subsample of participants during the second funding phase will be pooled to increase power for data analysis and to meet sample heterogeneity. Further, the larger sample size will enable us to conduct subgroup analyses with respect to motivational stage, activity behavior, and social engagement.

All participants underwent a short version of the Mini Mental State Examination during their first weekly group meeting [33]. Motivational stages to engage in recommended PA (endurance, strength, flexibility, and balance training), social-cognitive predictors for behavior change, and psychosocial factors (eg, quality of life, depression) were assessed in the self-administered questionnaire (for further detail on the instruments included in the questionnaire, see Table 1).

**Table 1:** Measures assessed in the self-administered study questionnaire.

Outcome measure		Instrument/scale	Time of assessment
**Physical activity**			
	Physical activity	Godin Leisure-Time Exercise Questionnaire (modified) [34]	T0^a^, T1^b^, T2^c^
	Recommended endurance, strength, balance, and flexibility training at follow-up	Self-generated items	T1, T2
	Stage of change regarding physical activity (endurance, strength, and balance + flexibility training, respectively)	HAPA^d^, stage of change, 3 items [35,36]	T0, T1, T2
	Intention to engage in physical activity	HAPA, intention, 2 items [36-38]	T0, T1, T2
	Self-efficacy regarding physical activity	HAPA, self-efficacy, 5 items [36,38]	T0, T1, T2
	Positive and negative outcome expectancies regarding physical activity	HAPA, outcome expectancies, 4 items [36,38,39]	T0, T1, T2
	Planning for physical activity	HAPA, planning, 6 items [38,40]	T0, T1, T2
	Habit strength regarding physical activity	Self-Report Habit Index, 2 items [41]	T0, T1, T2
	Physical self-description	PSDQ^e^ [42]	T0, T1, T2
**Physical environment**			
	Physical activity and neighborhood environment	Physical Activity Neighborhood Environment Scale [43]	T0
	Walking environment	Neighborhood Scales, walking environment [44]	T0
**Social support, social activities**			
	Social support for engaging in physical activity	Activity-related support by family and friends (modified) [45,46]	T0, T1, T2
	Social activities	Florida Cognitive Activities Scale (modified) [47,48]	T0, T1, T2
**Health behaviors**			
	Subjective age	Difference score (perceived physical age - chronological age = subjective physical age) [49]	T0, T1, T2
	Subjective health status	WHOQOL-BREF^f^, 1 item [50,51]; SF-36^g^, 1 item [52]	T0, T1, T2
	Health-related quality of life	EQ-5D-3L^h^ [53,54]	T0, T1, T2
	Objective health	Diseases and medication use (modified) [55]	T0
	Falls	EFST^i^ (modified) [56]	T0, T1, T2
	Fear of falling	GFFM^j^ [57]	T0, T1, T2
	Diet	FFQ^k^ (modified) [58]	T0, T1, T2
	Alcohol consumption	AUDIT-C^l^ [59]	T0, T1, T2
	Smoking behavior	Smoking Behavior Questionnaire, 1 item [60]	T0, T1, T2
	Stage assessment of smoking behavior	HAPA, stage of change [35,36]	T0, T1, T2
**Quality of life and well-being**			
	Quality of life	WHOQOL-BREF, 1 item [50,51]	T0, T1, T2
	Depression	CES-D^m^ [61]	T0, T1, T2
**Previous experiences with technology**			
	Use of computers/smartphones/apps	Self-generated items	T0, T1, T2
	Technology commitment	Technology Commitment Scale [62]	T0, T1, T2
**Use of, acceptance of, and satisfaction with interventions**			
	Use and acceptance of various components of the interventions (website and printed material), attendance of the offered group sessions, and overall satisfaction with the interventions	Self-generated items	T1, T2
	Preference regarding intervention material at baseline (retrospective)	Self-generated item	T2
	Reasons for crossing over or not crossing over after 3 months	Self-generated items	T2

^a^Baseline.

^b^3-month follow-up.

^c^9-month follow-up.

^d^HAPA: Health Action Process Approach.

^e^PSDQ: Physical Self-Description Questionnaire.

^f^WHOQOL-BREF: World Health Organization Quality of Life-BREF.

^g^SF-36: Short Form 36.

^h^EQ-5D-3L: 3-level version of the EQ-5D.

^i^EFST: Elderly Fall Screening Test.

^j^GFFM: Geriatric Fear of Falling Measurement.

^k^FFQ: Food Frequency Questionnaire.

^l^AUDIT-C: Alcohol Use Disorders Identification Test Short Version.

^m^CES-D: Centers for Epidemiologic Studies Depression Scale.

Anthropometric information, as self-reported in the questionnaire, included height in cm (only T0) and weight in kg (all time points). Sociodemographic information was assessed at baseline only, using items of the German Health Interview and Examination Survey for Adults [63] for date and country of birth, gender, native language, family and relationship status, living situation, education, employment status, and monthly net household income. Employment was assessed using one item of a questionnaire for assessing seniors’ demographic and sociostructural data in Germany [64]. In addition, the self-administered questionnaire at T1 and T2 includes self-generated items regarding adherence to the PA recommendations (weekly minutes of endurance, balance, and flexibility training and weekly units of muscle strength training per muscle group) and attendance of the offered group sessions. Furthermore, use and satisfaction with the intervention material and content are assessed with the following self-generated items: frequency of general use; use of different intervention components reporting using a 5-point Likert scale from “never” to “daily”; perceived helpfulness of intervention components, intervention content, and structure of weekly meetings rated on a 5-point Likert scale ranging from “not helpful at all” to “very helpful”; potential knowledge gained and perceived benefits of PA using “yes” and “no” response options; and whether participants would recommend the intervention to family and friends, answered using open-ended questions. Data on the reasons for dropping out of the study and the reasons for crossing over or not crossing over to the respective other intervention group were also collected.

### Interventions

To simplify and further develop the IT-based intervention implemented during the first funding phase and to translate it into a print-based intervention, 9 focus groups consisting of former participants of the previous intervention (n=1), other members of the target group (n=32), and stakeholders in communities such as members of senior citizen organizations and advisory boards (n=12) were held in May and June 2018. Participants of these focus groups were recruited via a press release and already established contacts to the stakeholders. Focus group discussions consisted of two parts. First, intervention materials used in the previous study and materials and health brochures for PA promotion developed by other researchers; federal agencies, such as the Federal Ministry of Health (BMG); or health insurance agencies were discussed with regard to their suitability for PA promotion in inactive older adults. In this context, the participants were invited to vote for the material they liked most and to justify their decision. Second, using the World Café method, participants were asked to discuss the following questions in small groups of 4-5 participants:

1. From your perspective, what are the key aspects of healthy aging?
2. Which topics related to healthy aging should be addressed in an intervention in order to reach physically inactive older adults?
3. What do inactive older adults need to be able to successfully participate in a PA intervention?
4. Which barriers need to be overcome and what would be helpful to these participants?
5. What would be motivating for inactive older adults for maintaining a healthy lifestyle?

The focus group discussions and votes for or against certain intervention materials were protocolled by the moderating researchers, and the resulting recommendations and requirements for the intervention material were applied when refining the existing IT-based intervention and when translating the IT-based intervention into a print-based intervention. Results consisted of recommendations regarding font size and color, amount of text to read, appropriateness of content (eg, inclusion of recommendations regarding diet, sleep hygiene, or pain management), and considerations with regard to the target group (eg, mobility, physical limitations, and the role of social support, loneliness, and wellbeing).

Both interventions are based on self-regulation theory [65,66] and principles of behavior change (eg, shaping knowledge, feedback and monitoring, goals and planning, social support, comparison of behavior, rewards) [67] and are designed to facilitate a physically active lifestyle by promoting regular self-monitoring of PA. Participants of both the IT-based and print-based interventions received PA recommendations based on WHO recommendations for this age group regarding endurance training (at least 150 minutes per week of moderate exercise, at least 75 minutes of intensive exercise, or a combination of both, in bouts of at least 10 minutes), strength training (at least 2 units per week for the 8 main muscle groups on non-consecutive days), and balance and flexibility training (at least 4 units per week for 5 minutes) [2]. Depending on gender, participants were provided with different brochures (online and offline) outlining exercises for different difficulty levels and displaying pictures of male or female older adults modeling these exercises.

**Figure 2:**
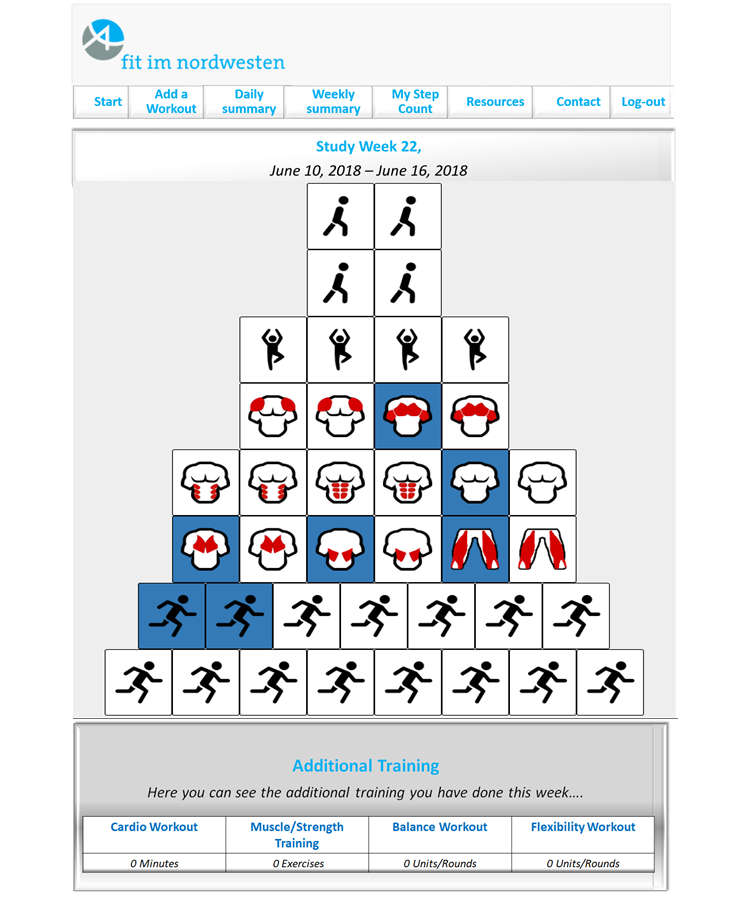
Weekly overview of physical activity performed, as provided to participants in the IT-based intervention group.

They also received a weekly PA diary in the form of a pyramid to subjectively track their PA behavior. Intervention material was printed for the print-based intervention group, while participants in the IT-based group received access to the exercise brochure and diary on a website and were provided the opportunity to download a smartphone app containing similar features. On the website or Android app, as well as in the printed diary, weekly feedback on whether participants reached PA goals was provided by displaying the amount of minutes or units exercised in the according week, as well as units required to reach the goal (ie, WHO recommendations for moderate exercise time, flexibility, and strength training). In addition to the weekly feedback, the website and Android app also provided participants a daily overview of PA. In the daily overview, participants had access to more detailed information about the exercises for the individual muscle groups, whereas in the weekly overview (in the IT-based and print-based interventions), only information about the progress towards the weekly goals was provided (see Figure 2). The app also allowed participants to record exercises without an active internet connection and synchronized recordings with the website, once the device was connected to the internet again. Participants in the IT-based intervention group who used the PA tracker in addition to the website or app also monitored their PA behavior objectively via synchronization of data regarding daily steps with the website. Data from the PA trackers will be analyzed based on methods presented in Meyer et al [68] to gain insights into participants’ PA behavior in everyday life during the time between assessments.

Paralleling the 10-week IT-based and print-based interventions, weekly group sessions with up to 25 participants per group were offered separately to both intervention groups. Trained student assistants led these sessions in which participants performed endurance, strength, balance, and flexibility exercises in groups or went for walks in the communities. Each session was 90 minutes long and designed to include 60 minutes of exercise, 10 minutes of answering questions, and 20 minutes of discussion of topics surrounding healthy aging that changed every week (eg, social support, relaxation, pain management). During their first weekly group meeting, participants received the necessary equipment (printed material or information to access the website and/or a Fitbit) and a comprehensive introduction on how to use the equipment and materials. After the first follow-up, interventions continued for another 6 months. After the initial 10 weeks of the interventions, the weekly group meetings were replaced with monthly events held over the course of the following 6 months. During these events, participants of all intervention groups were offered workshops and lectures on lifestyle-related topics, such as healthy nutrition in older age, overcoming loneliness, and strategies for developing healthy habits.

We will develop a toolbox during this project that will include both IT-based and print-based interventions and all related intervention materials that can be used later for long-term implementation and dissemination via different stakeholder groups (for further detail, please see the Discussion).

### Ethics Statement and Consent

Ethical approval was obtained from the Medical Association in Bremen (RA/RE-635, on July 3, 2018). The study was registered with the German Clinical Trials Register on January 10, 2019 – number DRKS00016073. All study participants were fully informed about the study and were requested to give informed consent.

## Results

In this study, we refined the previously developed IT-based intervention and translated it into a simple print-based intervention during the development phase from February 2018 to December 2018. The results of the qualitative focus groups held in May and June 2018 were analyzed and used to refine the existing intervention and to develop the print-based intervention. During the implementation phase of the study (baseline: January to April 2019; 3-month follow-up: April to July 2019; 9-month follow-up: September 2019 to January 2020), we expect to observe significant increases in PA at the 3-month follow-up after participation in both the print-based and IT-based interventions, in comparison with baseline, and a larger increase in PA in the 2 intervention groups than in the delayed-intervention control group of the preceding study (PROMOTE I).

We further expect that by providing some degree of choice to study participants 3 months into this study, we will be able to reduce loss to follow-up and improve long-term program adherence, when compared with the previous study. Further, we assume that participants with a ≥25% increase in PA level from baseline to the end of the follow-up period in the pooled sample across the 2 funding phases (ie, PROMOTE I sample and the subsample in PROMOTE II) will display significant improvements in indicators for healthy aging, such as cognitive function. Data collection was completed on January 31, 2020. Data cleaning and analysis started in February 2020. We expect to publish the first results of the study by the end of the funding period (January 2021).

## Discussion

This study will provide answers regarding the acceptance and effectiveness of IT-based vs print-based interventions for promoting uptake and maintenance of regular PA in initially inactive individuals aged 60 years and older. Further, we hope to generate the first results regarding the role of individual preferences for various intervention delivery modes in this study and the potential of a preference-based crossover design. In addition, this study will provide insights into the needs and demands of vulnerable groups (ie, inactive older adults). We will be able to identify “user groups” with regard to an affinity for specific intervention components.

This topic is of particular relevance in Germany, as 29% of the population is projected to be older than 65 years by 2030 [69]. Regular PA is a key contributor to healthy aging. Results regarding the effects of the interventions on PA and other health outcomes, such as quality of life, in initially inactive older adults over a relatively long period and their preferences for different intervention modes will be valuable to various stakeholder groups. For example, the work of stakeholders actively involved in community-based networks and senior citizen associations promoting population-based strategies for active aging will be informed by our results and implementation experiences. Further, the Prevention Law, which was passed in 2016 in Germany [70], mandated that health insurance agencies invest in health promotion and primary prevention in various contexts, including communities. Thus, our results will improve the existing evidence base on the effectiveness and implementation of community-based interventions in Germany and will support efforts to anchor evidence-based PA interventions in community structures and organizations via an allocation of permanent health insurance funds. To facilitate the rollout of the interventions, a toolbox for these stakeholder groups will be developed that includes PA assessment and monitoring tools allowing individuals to track their PA and helping health care professionals support their clients’ behavior change. We will also provide strategies to facilitate behavior adoption and maintenance and instructions to use personalized digital tools as standalone interventions without face-to-face assistance.

Last, complementing existing health and preventive care with eHealth intervention approaches, such as mobile apps for PA promotion, is in line with the current eHealth initiative of the Federal Ministry of Health [71]. The project is embedded in multidisciplinary research aimed at better understanding how changes in individual behavior and the environment and technology can help promote healthy aging. Our findings will inform the future development of complex interventions combining both local and regional policy changes aimed at promoting environmental changes and technology-based interventions targeting individual behavior change. Our experiences regarding this population’s data protection concerns will be communicated to policy makers currently involved in the eHealth initiative and will support the identification of suitable approaches to deal with these concerns.

Despite the advantages of the study design and objective PA measurement, several limitations can be identified. First, this study design does not include a control group, which might limit the interpretations that can be drawn from the potentially positive intervention effects. Second, the target population consists of inactive older adults, which are difficult to recruit. Third, the retrospective assessment of the preference for a certain intervention delivery mode, which could result in recall bias. However, we chose to retrospectively assess preferences because we anticipated disappointment (and possibly dropouts) if a person was not randomized to their preferred intervention group at baseline.

To conclude, this study will provide insights into the acceptance and effectiveness of an IT-based vs a print-based intervention for the promotion of PA in initially inactive individuals aged ≥60 years. In addition to answering the main research questions, we expect to obtain a better understanding of the interactions between numerous contextual factors and PA in this population. Results from this study will inform the work of various stakeholder groups actively involved in PA promotion at the population level and in different contexts and will support the shaping of new policies regulating the future design and implementation of preventive eHealth interventions for active aging.

## Appendix

Multimedia Appendix 1 Peer Review Report - AEQUIIPA.

## Abbreviations

AEQUIPA: Physical Activity and Health Equity: Primary Prevention for Healthy Ageing
AUDIT-C: Alcohol Use Disorders Identification Test Short Version
CES-D: Centers for Epidemiologic Studies Depression Scale
eHealth: electronic health
EFST: Elderly Fall Screening Test
EQ-5D-3L: 3-level version of the EQ-5D
FFQ: Food Frequency Questionnaire
GFFM: Geriatric Fear of Falling Measurement
HAPA: Health Action Process Approach
IT: information technology
MVPA: moderate-to-vigorous physical activity
PA: physical activity
PSDQ: Physical Self-Description Questionnaire
SF-36: Short Form 36
WHO: World Health Organization
WHOQOL-BREF: World Health Organization Quality of Life-BREF
