# Supplementary material for: Implementation and Effects of Information Technology-Based and Print-Based Interventions to Promote Physical Activity Among Community-Dwelling Older Adults: Protocol for a Randomized Crossover Trial
Source: JMIR Res Protoc. 2020 Apr 27;9(4):e15168. doi: 10.2196/15168 (PMC7215507; doi:10.2196/15168)
Supplement: Multimedia Appendix 1 [file resprot_v9i4e15168_app1.pdf]

Anlage:

Detaillierte Bewertung der Projektskizze

Proposal_ID	Acronym	Coordinator
004	AEQUIPA II	ZEEB

Promoting physical activity (PA) among the elderly is of high relevance for public health policy and practice. While the strategies to promote PA become better understood, this knowledge is not yet systematically implemented. By bringing together scientists from different disciplines including public health, epidemiology, gerontology, health psychology, urban planning, information technology and sports science, the AEQUIPA consortium can contribute to bridging this gap. The focus of the different subprojects on both structural/environmental aspects of PA among the elderly as well as on individual behavior change methods, while also accounting for equity and social status, makes the project particularly innovative. The project draws on relevant theory, with the socioecological model serving as an overall framework, and community mobilization, participatory intervention planning, trans-theoretical model of behavior change and self-regulation theory informing the sub-projects. Overall, it has a sound empirical component with adequate and state-of-the art methodology for performing community surveys, intervention trials and community-based intervention development. The consortium structure and management has been well thought through and the subprojects are well integrated into the overall research endeavor.

The interim report shows that coordination structures and communication within the consortium have been successfully set up, and networking with other national and international prevention research networks has been initiated. Strong links with local governments have also been established. The financial expenditure seems well managed and under control. The objectives for the first period have generally been met as planned and good progress has been made in the subprojects contributing to the main objectives, although some adjustments had to be made. While some difficulties that were encountered (e.g., willingness of older people to engage in PA activities) could have been anticipated, the proposed changes to remedy these problems are timely and adequate. The scientific output is very good, with 3 articles published and another 7 submitted to peer-reviewed journals, as well as 2 book chapters and several contributions to scientific meetings, although the latter were mainly at German symposia rather than international.

The Consortium's intention for the second phase to enhance dissemination, further strengthen a participatory approach in the development and implementation of PA promotion, and more systematically include external evidence from international studies and intensify the collaboration with other national prevention networks and researchers can be expected to yield a solid scientific output of the network. However, it remains to be proven if the approach proposed will be truly participatory, as it appears to remain very research-driven. The financial plan for stage 2, as detailed for the different subprojects, is well detailed and seems very reasonable.

Recommendation: The proposal is recommended for funding. For Subproject 3 the participatory nature of the intervention should be ascertained. With regard to subproject 7, it is noted that some workshops deal with generic research skills (meta-analysis, scientific writing) that are already taught at university and should not receive additional funding through this program.
